# Alginate-Based Microparticles Containing Albumin and Doxorubicin: Nanoarchitectonics and Characterization

**DOI:** 10.3390/ijms262411800

**Published:** 2025-12-06

**Authors:** Magdalena Kędzierska, Katarzyna Sala, Dominika Wanat, Dominika Wroniak, Magdalena Bańkosz, Piotr Potemski, Bożena Tyliszczak

**Affiliations:** 1 Department of Clinical Oncology, Central Clinical Hospital of the Medical University of Lodz, Pomorska 251, 92-213 Lodz, Poland; agdalena.kedzierska@umed.lodz.pl (M.K.); piotr.potemski@umed.lodz.pl (P.P.); 2 Department of Materials Engineering, Faculty of Materials Engineering and Physics, CUT Doctoral School, Cracow University of Technology, 37 Jana Pawla II Av., 31-864 Krakow, Poland; katarzyna.sala@doktorant.pk.edu.pl (K.S.); magdalena.bankosz@pk.edu.pl (M.B.); 3 Center for Advanced Functional Materials and Biomimetic Technologies, Warszawska 24, 31-155 Cracow, Poland; 4 Department of Materials Engineering, Faculty of Materials Engineering and Physics, Cracow University of Technology, 37 Jana Pawla II Av., 31-864 Krakow, Poland; dominika.wroniak@student.pk.edu.pl

**Keywords:** alginate microcapsules, drug delivery, biomedical applications

## Abstract

Alginate-based microcapsules have gained considerable attention as drug delivery systems due to their biocompatibility, biodegradability, and ability to encapsulate therapeutic agents. In this study, microcapsules were synthesized by crosslinking with calcium ions, with albumin serving as a carrier for doxorubicin. The goal was to develop a stable system capable of controlled drug release under physiological conditions, with potential applications in cancer therapy. Sodium alginate was used as a base polymer, which formed a stable matrix after crosslinking with calcium ions. The resulting microcapsules showed a uniform size distribution in the microscale range. Analyses confirmed their stability in simulated physiological environments with minimal degradation. Observations revealed a homogeneous structure of the microcapsules, while incubation studies confirmed controlled drug release triggered by pH changes. The results indicate that alginate–albumin microcapsules can serve as an effective platform for drug delivery, especially in cancer therapy and other biomedical applications.

## 1. Introduction

Modern drug delivery systems are continuously evolving to enhance the therapeutic efficacy and bioavailability of active compounds. Among various polymer-based carriers, alginate-based microcapsules have garnered significant interest due to their biocompatibility, biodegradability, and tunable properties, making them suitable for controlled drug release applications [1]. Wani et al. conducted a comprehensive review on the applications of chitosan- and alginate-based microcapsules in drug delivery systems. Their research has shown that appropriate modification of the polymeric structure allows precise control of the release rate of active ingredients, which may find applications in targeted anticancer therapy and other biomedical fields [2]. A key challenge in the development of effective microcapsule-based drug delivery systems lies in ensuring their potential application under physiological conditions, as it determines the duration of drug retention and eventual elimination from the body. Considering the available research on alginate drug delivery systems, it is worth noting the work of Li et al., who analyzed the effect of alginate structure on the stability of microcapsules and their ability to control doxorubicin release. They showed that increased alginate content in the matrix significantly reduced the rate of capsule degradation and delayed drug release. These results indicate that the degree of crosslinking of alginate with Ca^2+^ ions may be crucial for doxorubicin control. It is also worth referring to their analysis of the effect of the ratio of alginate to drug on the pore size of microcapsules, which directly translates into the rate of release of the active ingredient. These observations indicate that the amount of albumin in the system can directly control the release kinetics of doxorubicin and the stability of the capsules in a biological environment [3].

Albumin, a naturally occurring protein with well-established drug-binding properties, has been explored as a carrier to enhance the stability and biocompatibility of alginate-based microcapsules, particularly in biomedical applications [4,5]. In a study by Elzoghby et al., it was shown that albumin can be effectively used to create protein nanoparticles that improve the stability and controlled release of drugs [6,7]. By incorporating albumin into alginate-based microcapsules, the physicochemical properties of the system can be optimized, potentially improving its integrity and performance in biological environments [8]. Albumin also plays a crucial role in modulating the microcapsule surface properties, which may affect interactions with cells and biological fluids, further influencing their therapeutic efficacy.

Previous studies have demonstrated the effectiveness of alginate microcapsules for encapsulating a wide range of bioactive compounds, including peptides, proteins, and small-molecule drugs [9]. In particular, doxorubicin-loaded alginate microcapsules have been investigated as a potential tool for localized and sustained release in cancer therapy, addressing the limitations associated with conventional systemic administration [10]. Additionally, alginate-based carriers have been studied for their ability to protect encapsulated drugs from premature degradation, enhancing their stability and bioavailability. The physicochemical properties of the microcapsules, such as size distribution, porosity, and surface charge, play a crucial role in determining their interaction with biological fluids [11]. Furthermore, the incorporation of albumin as a stabilizing agent in these microcapsules may influence their mechanical properties and degradation rate, factors that are essential for ensuring the controlled and sustained presence of the encapsulated therapeutic agents in targeted tissues [12]. The presence of albumin can also impact the hydration and swelling behavior of the microcapsules, which may contribute to their stability and retention in biological environments. The structural integrity of alginate microcapsules under physiological conditions is a critical factor affecting their potential biomedical applications. Studies have shown that the crosslinking density of the alginate matrix, influenced by calcium ion concentration and gelation conditions, directly impacts the microcapsules’ resistance to dissolution and mechanical stress [13]. Additionally, environmental factors such as pH and ionic strength can modulate the stability and swelling behavior of the microcapsules, further affecting their performance in drug delivery applications [14]. The degradation behavior of alginate microcapsules in simulated physiological fluids is particularly relevant for applications in controlled release systems. Moreover, albumin incorporation may alter the microcapsules’ degradation profile by interacting with calcium crosslinks and modifying the gel network, potentially providing additional control over their stability and longevity in biological environments. These factors collectively underscore the importance of understanding the interplay between material composition, structural properties, and environmental conditions to design optimized alginate–albumin microcapsules for biomedical applications. Future research should focus on further characterizing the mechanical resilience, biodegradation patterns, and interactions with biological tissues to refine these microcapsules as a viable platform for targeted therapeutic delivery.

In this study, alginate-based microcapsules incorporating albumin and doxorubicin were synthesized using ionic gelation with calcium ions. The primary objective was to evaluate their physicochemical properties, structural stability, and response to simulated physiological conditions to determine their potential as controlled drug delivery systems. The microcapsules were extensively characterized through multiple analytical techniques, including weight loss analysis to assess degradation kinetics, incubation in simulated body fluids to mimic in vivo conditions, microscopic observations to evaluate morphological stability, and physicochemical analysis using FT-IR spectroscopy to confirm structural integrity and potential interactions between alginate, albumin, and doxorubicin. A key novelty of this study lies in the incorporation of albumin within the alginate matrix as both a stabilizing and functionalizing agent. Unlike conventional alginate–doxorubicin carriers, which primarily rely on ionic crosslinking to achieve stability, the inclusion of albumin introduces additional structural reinforcement, potentially reducing premature drug leakage and enhancing biocompatibility. Our approach emphasizes the structural integrity and environmental adaptability of microcapsules under simulated physiological conditions. By evaluating their response to different biochemical environments, this study provides a deeper understanding of how albumin affects the degradation and stability of microcapsules within parameters critical to ensuring sustained drug release and minimizing systemic toxicity. The results obtained contribute to the growing body of research on biopolymer-based drug delivery systems and provide valuable insights into the design of advanced carriers for targeted cancer therapy.

## 2. Results and Discussion

### 2.1. Analysis of Weight Loss

**Figure 1:**
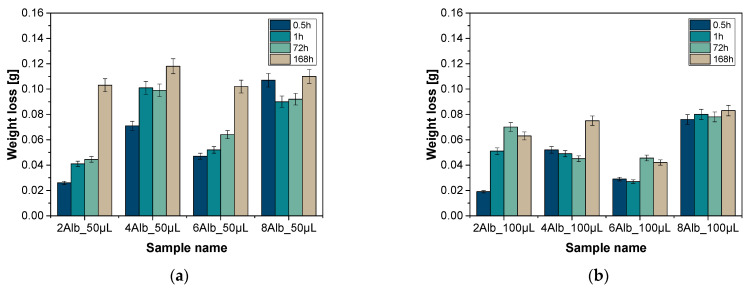
Results of weight loss in distilled water containing 50 mL (**a**) and 100 mL (**b**) of a mixture of sodium alginate with albumin and doxorubicin, respectively.

**Figure 2:**
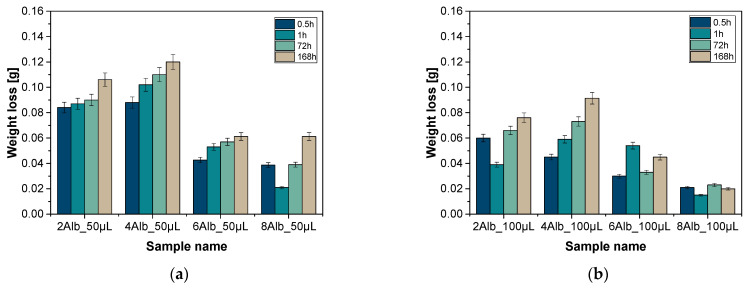
Results of weight loss in PBS solutions containing 50 mL (**a**) and 100 mL (**b**) of a mixture of sodium alginate with albumin and doxorubicin, respectively.

Weight loss analysis was carried out to determine and compare the degree of degradation of microcapsules depending on the content of albumin in their composition. The study made it possible to assess the structural stability of the carriers under different conditions and the effect of albumin on their degradation. The results of the analysis for PBS liquid and distilled water are shown in the graphs in Figure 1 and Figure 2.

The test results showed significant weight loss for all samples analyzed, with the greatest weight reduction occurring 0.5 h after the capsules were immersed in the solution. This effect was particularly pronounced for the series containing 100 mL of a mixture of sodium alginate with albumin and doxorubicin, suggesting a greater susceptibility of these carriers to degradation during the initial phase of contact with the medium. A similar development was observed in a study by Zhang et al., where biodegradable starch-alginate films in the presence of montmorillonite showed rapid initial weight loss, indicating that the polymer structure has a significant effect on initial degradation in aqueous environments [15]. This may be due to specific structural properties of the capsules, such as porosity, the degree of crosslinking with calcium ions and the distribution of albumin in the matrix. A correlation between capsule size and mass loss was also observed—carriers with smaller diameters were characterized by a more intensive degradation process, which may suggest their greater suitability for applications requiring faster modification of the capsule structure in a biological environment. Niu et al. showed that the combination of sodium alginate with chlorogenic acid can affect the rate of hydrogel degradation, regulating inflammatory processes and intestinal microflora, suggesting the potential use of alginate microcapsules in the treatment of inflammatory diseases [16]. On the other hand, the smallest weight loss was found in the 6Alb_50mL/6Alb_100mL and 8Alb_50mL/8Alb_100mL samples in both analyzed series immersed in PBS solution, which may indicate an excessive amount of albumin in their composition, leading to increased structural stability of the carriers. The structural stability of alginate microcapsules under physiological conditions is a key factor affecting their potential biomedical applications. Studies by Lee et al. have shown that the stability of alginate microcapsules under in vivo conditions depends on the alginate composition and crosslinking conditions, which affects their resistance to enzymatic and mechanical degradation [17]. In contrast, Wang et al. confirm in their study that the introduction of silk fibroin into alginate composites can control their degradation, indicating that appropriate composition modifications can affect the stability of materials under biological conditions [18].

An interesting phenomenon was the small increases in mass recorded after the initial period of intensive degradation, which may be the result of capsule swelling due to absorption of the medium. This phenomenon was more noticeable in PBS solution, indicating a significant influence of the environmental composition on the structural behavior of the media. In addition, analysis of the changes in color and turbidity of the medium revealed that in the PBS solution, after just 1 h, the solution turned from transparent to pink, and the samples themselves transformed from purple to orange. Similar degradation mechanisms of alginate carriers have been described by He et al., who studied alginate platforms as carriers for cancer therapy [19]. The intensity of these changes was clearly greater for the batch containing 100 mL of the aforementioned mixture, suggesting faster degradation processes in this group. After one week, all the media in PBS were completely discolored. The changes in the color of the microcapsules could be the result of polymer degradation processes, the release of substances contained in them or interaction with the surrounding environment. In samples immersed in distilled water, these processes were much slower—no significant changes were observed for the first three days, and after a week, only slight color modifications were visible.

### 2.2. Incubation in Simulated Body Fluids

**Figure 3:**
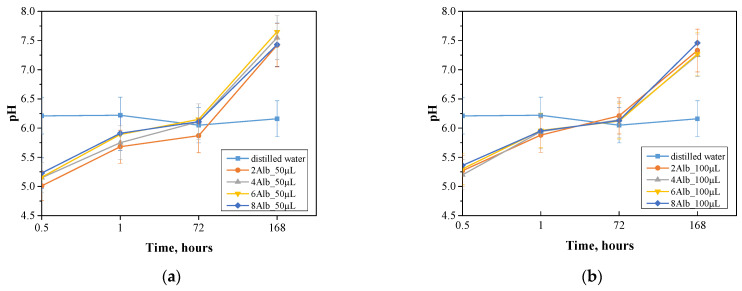
Changes in pH during incubation of samples in distilled water containing 50 mL (**a**) and 100 mL (**b**), respectively, of a mixture of sodium alginate with albumin and doxorubicin.

**Figure 4:**
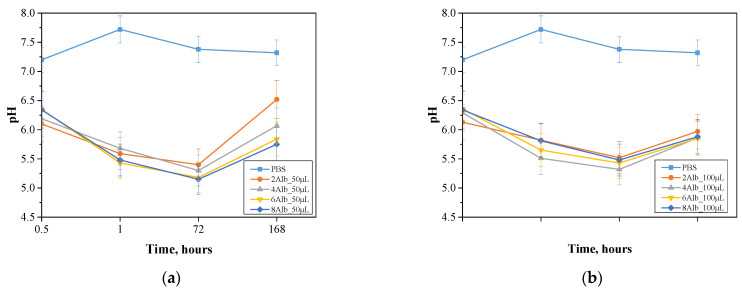
Changes in pH during incubation of samples in PBS solution containing 50 mL (**a**) and 100 mL (**b**) of a mixture of sodium alginate with albumin and doxorubicin, respectively.

The incubation study was conducted to measure the pH, assess the stability and degradation of albumin capsules in which the prepared dried spherical protein carriers were incubated for 0.5, 1, 72 and 168 h, respectively. The results obtained are shown in the Figure 3 and Figure 4.

The stability of albumin microcapsules is strongly dependent on the pH value of the surrounding environment, which directly affects their degradation and structural integrity. A similar pH-dependent behavior was noted by Ji et al. when studying empty pectin-based nanocapsules, where changes in pH affected drug release and stability [20]. High pH promotes the process of denaturation of proteins, which can lead to a decrease in the effectiveness of the capsules as carriers, while in an acidic environment, intense hydrolysis is observed, accelerating their degradation. A comparable trend was observed in a study by Xu et al. on polymer-functionalized silica nanoparticles, where pH conditions modulated the release profile of quercetin, demonstrating the critical role of environmental pH in drug carrier performance [21]. The study showed that the PBS solution has greater stability compared to distilled water, as evidenced by smaller fluctuations in pH values. Distilled water tends to have a gradual increase in pH, which may suggest an interaction of the capsule components with the medium, leading to changes in its chemical properties. The largest differences in pH values over the course of the study were observed for the batch containing 50 mL of a mixture of sodium alginate with albumin and doxorubicin, which may indicate a more intensive degradation process of the capsules and their greater susceptibility to breakdown under changing environmental conditions. This may also indicate greater diffusion of the capsule components into the medium, which for biomedical applications suggests faster charge release in response to pH changes. In contrast, the lowest pH variation was observed for a batch containing 100 mL of a mixture of sodium alginate with albumin and doxorubicin, in PBS solution, suggesting high structural stability of these capsules. The lower pH variability may be due to the limited interaction of the capsules with the medium, indicating their increased resistance to degradation and limited diffusion of the components into the environment. This fact is consistent with the observation that increasing the size of the capsules decreases their susceptibility to degradation, which may be beneficial in systems that require a longer time for the carrier to act in the body. For samples incubated in distilled water, the size of the capsules showed no significant effect on the change in pH value, suggesting that the dominant factor affecting the stability of the system is not the size of the carrier, but its composition and the physicochemical properties of the medium.

The results of the analysis indicate that the stability of albumin microcapsules is strongly dependent on the environment in which they are placed. The PBS solution provides greater control over pH changes, which may be crucial in biomedical applications, while the 50 mL series shows greater susceptibility to degradation, which may suggest its use in systems requiring faster release of substances.

### 2.3. Microscopic Observations

**Figure 5:**
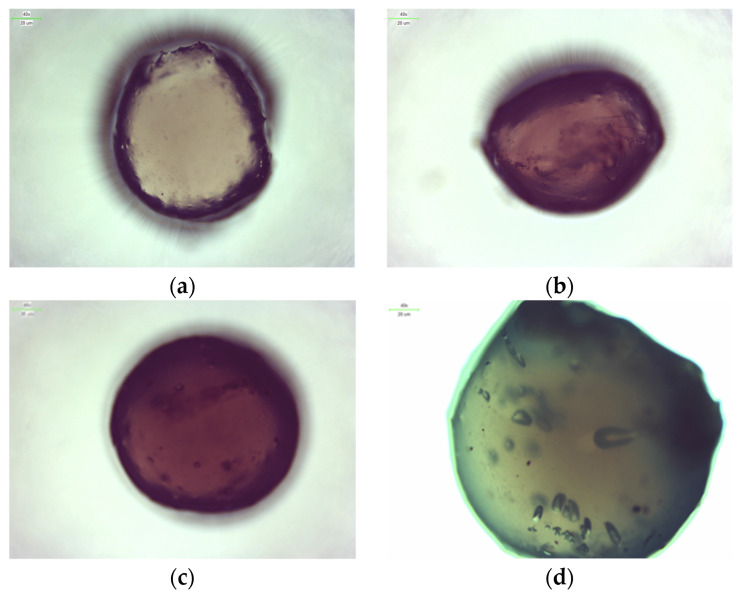
Images of nanoparticles from an optical microscope: 2Alb_50mL (**a**); 4Alb_50mL (**b**); 6Alb_50mL (**c**); 8Alb_50mL (**d**).

**Figure 6:**
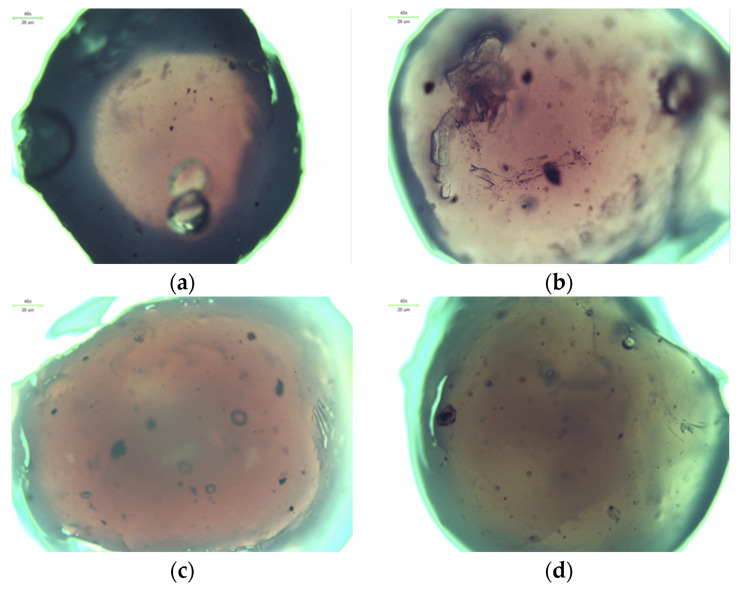
Images of nanoparticles from an optical microscope: 2Alb_100mL (**a**); 4Alb_100mL (**b**); 6Alb_100mL (**c**); 8Alb_100mL (**d**).

**Figure 7:**
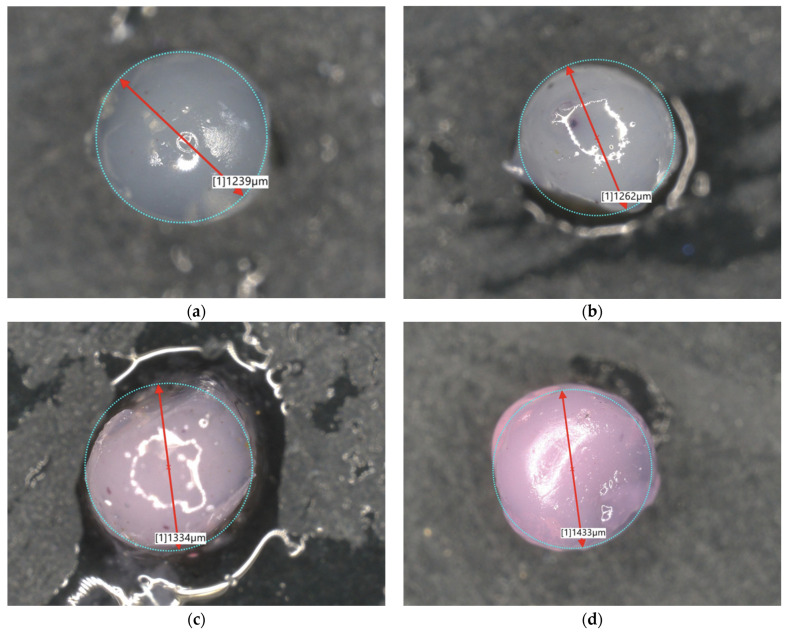
Images of nanoparticles from an digital microscope: 2Alb_50mL (**a**); 4Alb_50mL (**b**); 6Alb_50mL (**c**); 8Alb_50mL (**d**) (magnification ×500).

**Figure 8:**
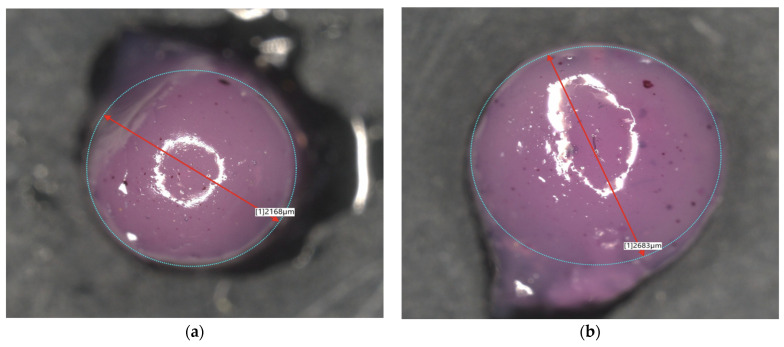

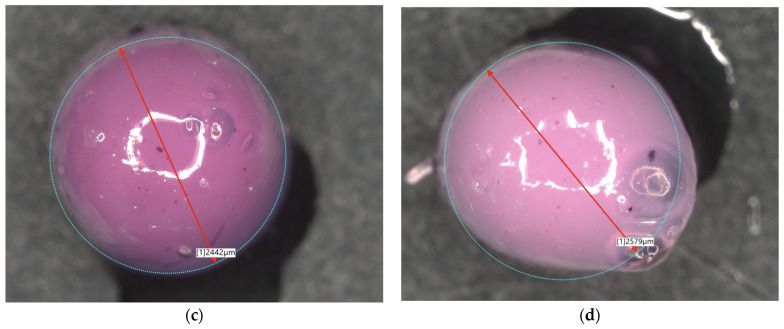
Images of nanoparticles from a digital microscope: 2Alb_100mL (**a**); 4Alb_100mL (**b**); 6Alb_100mL (**c**); 8Alb_100mL (**d**) (magnification ×500).

Observation using optical and digital microscopy was carried out to image and compare the surfaces of synthesized albumin carriers containing doxorubicin. Below, Figure 5 and Figure 6 are the images obtained by optical and digital microscopy, as are Figure 7 and Figure 8.

Structural studies of albumin microcapsules carried out by digital microscopy allowed an accurate assessment of their morphology and potential factors affecting degradation. The obtained images showed a homogeneous, spherical structure of the capsules with a smooth surface, which indicates their uniform organization after crosslinking with calcium ions. At the same time, the presence of small gas bubbles may indicate the uncontrolled introduction of air during the mixing process, potentially affecting the mechanical properties of the carriers and their stability in a liquid environment. Table 1 shows the sizes of each sample.

The diameters of the microcapsules from the 50 mL series (average ≈ 1.2 mm) and the 100 mL series (average ≈ 2.4 mm) confirm the high reproducibility of the synthesis method used, which is crucial for controlling their physicochemical properties, including degradation kinetics. Differences in capsule size are important for the rate of degradation, as the smaller capsules, which contained 50 mL of a mixture of sodium alginate together with albumin and doxorubicin, exhibit a larger specific surface area, which may contribute to more intense erosion of the material and accelerated weight loss compared to microcapsules containing 100 mL of the aforementioned mixture. In addition, the observed discoloration inside the capsules, resulting from incomplete dissolution of doxorubicin in distilled water before the encapsulation process, may indicate heterogeneous distribution of the substance in the protein matrix. Consequently, this may lead to local differences in the solubility and permeability of the capsule structure, thus affecting its gradual degradation. The lighter coloration of the 50 mL series microcapsules of the sodium alginate mixture suggests greater transparency resulting from their smaller size, which may correlate with more dynamic weight changes during the initial stages of incubation. In contrast, the larger, darker capsules of the 100 mL series of the alginate mixture may exhibit more controlled and slower degradation, indicating their potentially longer duration in drug delivery systems.

Microscopic analysis allows us to draw conclusions about the relationship between capsule size, homogeneity and mechanical stability, which directly translates into the degree of degradation in physiological environments. Light microscope observations showed that the microcapsules retained their spherical shape and integrity under physiological conditions. Similar morphological stability was demonstrated by Gao et al., who developed a nanoparticle formulation of paclitaxel bound to albumin with a high concentration of the drug, demonstrating that preservation of the spherical structure is crucial for prolonging circulation time and increasing bioavailability in biological systems [22]. These results underscore the importance of precise control of synthesis parameters, such as substrate mixing and crosslinking process, in order to obtain optimal functional properties of protein carriers.

### 2.4. Physicochemical Analysis

**Figure 9:**
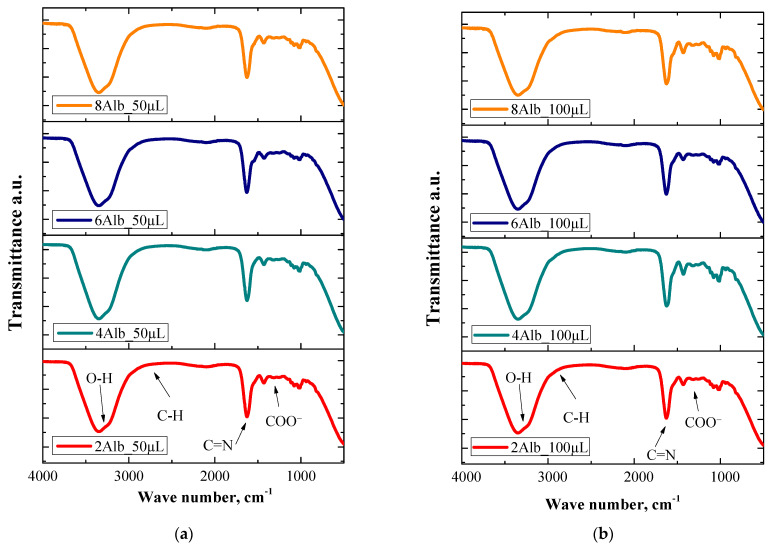
FT-IR spectroscopy spectra of alginate microcapsules for: 50 mL (**a**) and 100 mL (**b**) of a mixture of sodium alginate with albumin and doxorubicin, respectively.

The aim of this study was to analyze the chemical structure of various samples by FT-IR spectroscopy, enabling the identification of key functional groups and assessing differences due to differential albumin content. The obtained spectra of the microparticles are shown in Figure 9.

Based on the above graphs, the functional groups of the albumin capsules studied were identified. The absorption bands shown in the FT-IR spectra graphs are mainly derived from albumin. The wave number of 3420 cm^−1^ indicates the presence of an O-H stretching bond, while the band with a wave number of 1654 cm^−1^ corresponds to C=N stretching bonds. The size of the synthesized capsules for this analysis did not prove to be significant, as identical spectral bands were obtained for both the 50 mL and 100 mL batches. Additional characteristic peaks confirm the presence of functional groups associated with the components of the microcapsules. The absorption band around 2920 cm^−1^ can be attributed to asymmetric stretching vibrations of C-H bonds, which are characteristic of organic compounds such as proteins and polysaccharides. The peak observed at 1400 cm^−1^ corresponds to symmetric bending vibrations of carboxylate groups (COO^−^), indicating the presence of alginate as a key structural component. Furthermore, a distinct absorption band at approximately 1030 cm^−1^ suggests the presence of C-O-C stretching vibrations, which are typical for glycosidic bonds in polysaccharides [23]. The results indicate strong interactions between alginate, albumin, and doxorubicin within the microcapsules. The absence of significant shifts in peak positions between the different capsule sizes suggests that the encapsulation process does not alter the primary chemical structure of the components. Similar results were obtained in a study by Roacho-Pérez et al., who analyzed chitosan microspheres with magnetite using FT-IR analyses. They showed that the absence of significant shifts in the absorption bands after the introduction of magnetite indicates that the structural integrity of the chitosan matrix is preserved, which is consistent with our observations on alginate microparticles [24]. On the other hand, Srivastava et al. observed that the introduction of tartrate as a co-crosslinker into chitosan-based microcapsules changed the intensity of the absorption bands in the carboxyl groups, indicating changes in the crosslinking of the polymer. In the present study, the lack of significant changes in the 1400 cm^−1^ range confirms that ionic crosslinking with Ca^2+^ ions does not disrupt the alginate structure in the presence of albumin [25]. However, a slight broadening of the O-H stretching band at 3420 cm^−1^ may suggest hydrogen bonding interactions between albumin and alginate, which could contribute to the structural stability of the capsules. Additionally, the intensity of the amide I (1654 cm^−1^) and amide II (1540 cm^−1^) bands, characteristic of proteins, confirms the successful incorporation of albumin into the alginate matrix. A study by Steichen et al. on P((MAA-co-NVP)-g-EG) hydrogels showed that the intensity of these bands can correlate with the level of polymer-protein interaction. Our results indicate that albumin remains an integral component of the microparticle structure, which is consistent with their observations on the effect of proteins on hydrogel crosslinking [26].

The presence of characteristic peaks associated with doxorubicin is also notable. A small but identifiable absorption band at approximately 1730 cm^−1^ corresponds to C=O stretching vibrations from ester or ketone functional groups, confirming the presence of the drug in the system. The slight shifts and intensity changes in certain peaks, particularly in the amide and carbonyl regions, suggest possible interactions between doxorubicin and the albumin-alginate matrix, which may influence the drug release profile. FT-IR analysis confirmed the structural integrity of the synthesized microcapsules, as well as the presence of characteristic functional groups of their key components. The observed spectral features suggest effective encapsulation of albumin and doxorubicin within the alginate matrix, with potential intermolecular interactions that may enhance the stability and controlled release properties of the drug delivery system.

### 2.5. Doxorubicin Release Study

**Figure 10:**
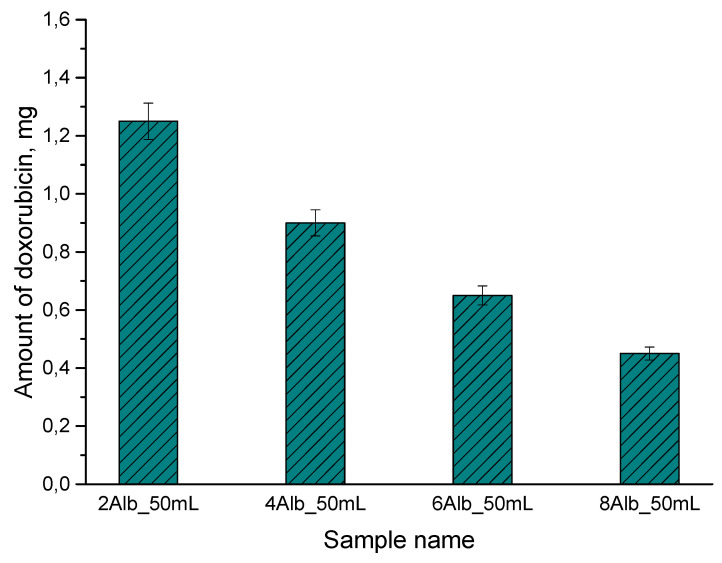
Amount of doxorubicin released after 24 h from alginate–albumin microspheres containing different concentrations of albumin (2Alb, 4Alb, 6Alb, 8Alb).

Figure 10 presents the quantitative analysis of doxorubicin released from the alginate–albumin microspheres after 24 h of incubation in PBS, illustrating the influence of albumin concentration on the efficiency of drug release.

Based on the obtained results, a clear relationship was observed between the concentration of albumin used during the synthesis of microspheres and the amount of doxorubicin (DOX) released into the medium after 24 h of incubation. All formulations contained the same initial DOX load (2.5 mg per batch), whereas they differed in the amount of albumin incorporated into the alginate matrix (2, 4, 6, and 8 mg/mL). The microspheres with the lowest albumin content (2Alb_50mL) exhibited the highest amount of released doxorubicin—approximately 1.25 mg. With increasing albumin concentration, a systematic reduction in DOX released into the medium was observed: approximately 0.90 mg (4Alb), 0.65 mg (6Alb), and 0.45 mg (8Alb). This demonstrates that higher albumin content significantly limits the availability of the drug for rapid diffusion. These observations arise from the intrinsic properties of albumin, which contains numerous drug-binding sites, including sites capable of interacting with doxorubicin. Electrostatic and hydrophobic interactions between DOX and albumin partially sequester the drug within the microsphere structure, reducing the fraction of free DOX able to diffuse quickly. Higher albumin concentrations also promote the formation of a denser internal network within the alginate hydrogel, further decreasing matrix permeability and slowing DOX transport into the release medium. Consequently, microspheres with the highest albumin content (8Alb) exhibited the lowest 24-h release level, indicating their potential for sustained drug retention and more controlled release. In contrast, the 2Alb microspheres showed the highest “burst release,” resulting from weaker DOX binding and a more porous matrix structure. In summary, the results clearly demonstrate that increasing albumin content in alginate microspheres enhances the retention of doxorubicin within the carrier structure and markedly reduces the amount of drug released during the first 24 h. This effect may be exploited in the design of delivery systems with tailored release profiles, depending on the desired therapeutic outcome.

Compared to alginate or albumin systems available in the literature for controlled drug release, the alginate–albumin–doxorubicin system developed in this work demonstrates several significant functional advantages, resulting from both the mechanism of matrix structure and its quantitative characterization. First, the obtained microcapsules were characterized by structural stability without significant geometric deformation, which is an advantage over the systems reported by Sun et al. [12], where maintaining morphology required the use of microfluidics. Our droplet method allowed for obtaining comparably homogeneous capsules, but in a much simpler technological manner and potentially easier to scale. Second, the results of degradation analysis showed a rapid but controlled mass loss within the first hour (up to ~25–30%), followed by stabilization over a longer incubation time, indicating regulated diffusion of the medium into the capsules. In the study by Zhang and Chen [15], a similar weight loss was observed, indicating a greater susceptibility of their systems to hydrolysis. Therefore, our system, thanks to the synergy of crosslinked alginate and albumin, is characterized by a more controlled degradation–swelling balance, and therefore higher physical stability in aqueous environments and PBS.

A key advantage of our solution is the ability to precisely model drug release by regulating the albumin content in the matrix. While alginate systems without added proteins, as in the work of Qiao et al. [3], obtained profiles primarily dependent on the degree of alginate crosslinking and pH, in the presented system, albumin plays an additional regulatory role, creating an additional “repository” of drug molecules through binding, limiting their rapid release. This mechanism is confirmed by quantitative differences: increasing albumin concentration resulted in a decrease in the amount of released drug. This is a significant effect, exceeding the scale of the burst release reduction described for pure alginate systems in He et al. [19], where changes in crosslinking parameters resulted in significant reductions. Furthermore, the obtained values are also more favorable than in classical albumin nanocarriers described by Elzoghby et al. [6], in which release control resulted primarily from slow nanoparticle degradation rather than modulation of the number of drug-binding sites. Therefore, incorporating albumin into the alginate matrix allows not only for slowing diffusion but also for active, molecular “blocking” of DOX through protein-drug interactions, which constitutes a mechanistic advantage over existing solutions.

## 3. Materials and Methods

### 3.1. Materials

Alginic acid in solid form was purchased from Pol-Aura (Dywity, Poland). PBS buffer (pH = 7.2–7.6) in tablet form and dexorubicin were purchased from Sigma Aldrich (Saint Louis, MO, USA). In turn, albumin (ovalbumin powder) and calcium chloride were purchased from Chempur (Piekary Śląskie, Poland).

### 3.2. Synthesis of Protein Carriers

**Figure 11:**
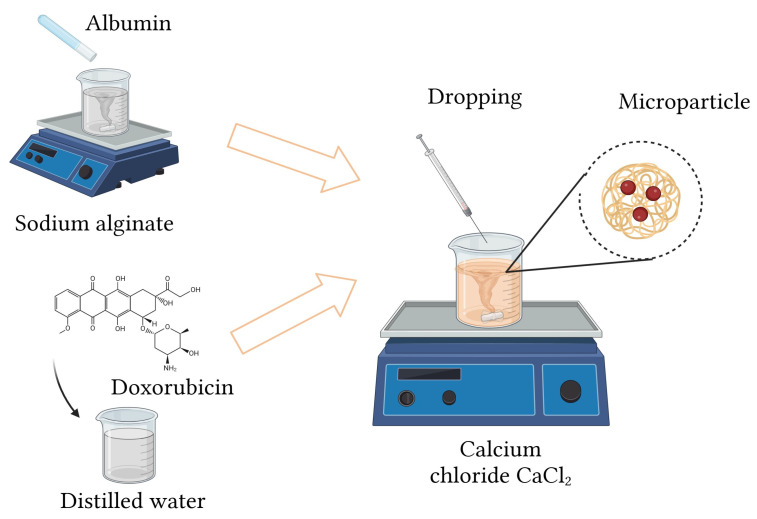
Scheme of obtaining microparticles.

The synthesis of protein capsules was carried out by preparing the necessary synthesis substrates. In the first step, a solution of sodium alginate was prepared using a magnetic stirrer RT IKA company. Albumin was introduced into the prepared sodium alginate solution as a protein component. Doxorubicin was dissolved in distilled water, which allowed its uniform distribution in the obtained reaction mixtures. These prepared mixtures were dropped into 50 and 100 mL of CaCl_2_ solution at a ratio of 25 g/100 mL of distilled water. The reaction took place at room temperature with continuous stirring using a magnetic stirrer. During the gelation step, calcium ions (Ca^2+^) diffuse into the alginate-containing droplets and induce ionic crosslinking by coordinating with the carboxylate groups of the alginate chains. This process results in the rapid formation of a three-dimensional hydrogel network that stabilizes the droplet structure. Albumin molecules become uniformly entrapped within the developing polymer matrix, while doxorubicin is incorporated through physical encapsulation and noncovalent interactions with both alginate and albumin. The final microparticles therefore consist of an ionically crosslinked alginate network with embedded albumin and distributed doxorubicin, as schematically represented in Figure 11.

By crosslinking with calcium ions, protein capsules of two diameter sizes, i.e., ≈1.2 mm and ≈2.5 mm, were obtained. A summary of the substrates used for the synthesis of protein carriers is shown in Table 2. The spatial distribution of alginate, albumin, and doxorubicin within the resulting microparticles, as well as the key stages of their formation, is illustrated in Figure 11 to provide a clearer representation of the interactions and structural organization occurring during the synthesis process.

**Figure 12:**
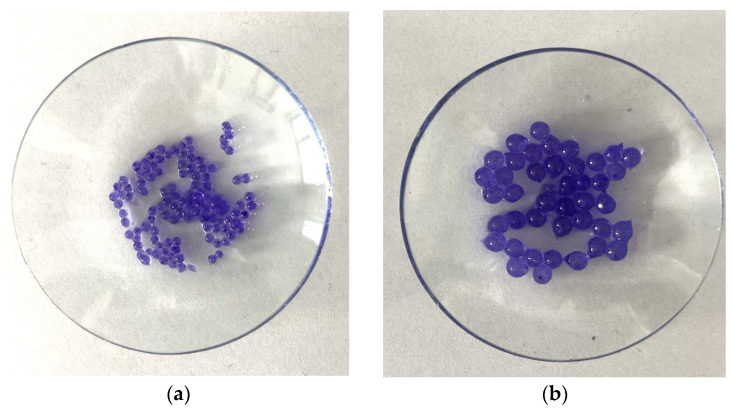
Sample photos: 50 mL (**a**) and 100 mL (**b**) of a mixture of sodium alginate with albumin and doxorubicin, respectively.

The resulting microcapsules were subjected to further physicochemical characterization. A scheme for obtaining and a photo of sample microcapsules are presented in Figure 11 and Figure 12.

### 3.3. Analysis of Weight Loss

Weight loss analysis focused on evaluating the properties of albumin containing therapeutic substance. The process began with the preparation and drying of the samples, which were then accurately weighed on a Radwag analytical balance. The samples were then placed in two different incubation fluids: phosphate-buffered saline (PBS) and distilled water. The samples were incubated for different time periods: 0.5 h, 1 h, 72 h and 168 h. After each incubation step, excess fluid was removed and the weight of the samples was measured again. Based on this, the weight loss rate was calculated using the equation:

mass loss = m_0_ − m

where

m—mass of swollen protein capsule, g;
m_0_—mass of dry protein capsule, g.

Each sample was subjected to four replicates, and the resulting weight loss values were averaged to obtain representative data.

### 3.4. Incubation in Simulated Body Fluids

In the next step of this study, microparticles were incubated in phosphate-buffered saline (PBS) to replicate the environment of the human body and evaluate the properties of the material under these conditions and in distilled water. The purpose of this experiment was to analyze the effect of incubation in PBS on changes in pH and ionic conductivity (ELMETRON, Zabrze, Poland), which may indicate ion exchange processes, material degradation or release of active substances from microparticles. This study was carried out over 168 h, with pH and ionic conductivity measurements taken at specific time points (0.5, 1 and 72 h). Calibrated pH and conductivity meters were used to ensure high precision of the results. Microparticle samples were placed in sterile containers filled with 40 mL PBS and incubated at 37 °C to simulate physiological conditions. Changes in pH were monitored to assess the release of ions or other substances from the microparticles that may affect the pH stability of the surrounding environment. At the same time, changes in ionic conductivity allowed evaluation of material degradation and the ion exchange process between microparticles and PBS. By performing regular measurements, a complete profile of these parameters over time was obtained, providing an assessment of the interaction between microparticles and physiological fluids, as well as their potential applications in biological environments.

### 3.5. Microscopic Observations

To further characterize the microparticles, a Keyence digital microscope was used, allowing precise observation of the surface structure and measurement of sample dimensions. The microparticles were observed under the microscope, and their diameters were accurately measured, allowing evaluation of the size distribution and uniformity of the capsules.

### 3.6. FT-IR Spectroscopy

As part of the physicochemical analysis, the materials obtained were evaluated by Fourier transform infrared (FT-IR) spectroscopy. The analysis was carried out using a Nicolet iS5 spectrometer (Thermo Scientific, Waltham, MA, USA). The spectra were recorded in the range of 4000–500 cm^−1^, with a resolution of 4.0 cm^−1^, under room temperature conditions. The FT-IR study made it possible to identify the functional groups present in the samples and evaluate the chemical interactions in the materials obtained.

### 3.7. Doxorubicin Release Study

The in vitro release of doxorubicin from the alginate–albumin microspheres was evaluated using UV–Vis spectrophotometry. Microspheres from each formulation (2Alb, 4Alb, 6Alb, 8Alb) were placed in individual containers and suspended in 50 mL of phosphate-buffered saline (PBS, pH 7.4). The samples were incubated at 37 °C under gentle shaking to simulate physiological conditions. After 24 h, a 3 mL aliquot of the release medium was collected for analysis. The DOX concentration was determined using a UV–Vis spectrophotometer at λ = 480 nm, corresponding to the maximum absorbance peak of doxorubicin. All measurements were performed in triplicate. Quantification was carried out using a previously prepared calibration curve in PBS within the linear range of 0–20 mg/L.

## 4. Conclusions

This study focused on the synthesis and characterization of polymeric material carriers containing albumin and doxorubicin. The results of the weight loss analysis showed significant weight loss for all the samples analyzed, with the greatest weight reduction occurring 0.5 h after the capsules were immersed in the solution. Microparticles with smaller diameters were characterized by a more intensive degradation process, which may suggest their greater suitability for applications requiring faster modification of the capsule structure in a biological environment. Sorption capacity tests confirmed that the PBS solution has greater stability compared to distilled water, as evidenced by smaller fluctuations in pH values. The largest differences in pH values over the course of the study were observed for the batch containing 50 mL of a mixture of sodium alginate with albumin and doxorubicin, which may indicate a more intensive degradation process of the capsules and their greater susceptibility to breakdown under changing environmental conditions. The lowest pH variation was observed for a batch containing 100 mL of a mixture in PBS solution, suggesting high structural stability of these capsules. The lower pH variability may be due to the limited interaction of the capsules with the medium, indicating their increased resistance to degradation and limited diffusion of the components into the environment. These observations confirm that the stability of albumin microcapsules is strongly dependent on the environment in which they are placed.

FT-IR analysis further confirmed the presence of key functional groups in the synthesized microcapsules, including characteristic peaks for O-H, C=O, and C-H bonds. These findings suggest strong interactions between alginate, albumin, and doxorubicin within the polymeric matrix, contributing to structural integrity and controlled drug release. The amide I and amide II bands confirm the successful incorporation of albumin, while the C=O stretching vibration around 1730 cm^−1^ supports the presence of doxorubicin. Additionally, slight shifts in peak positions indicate possible intermolecular interactions influencing the release profile.

Microscopic observations revealed that the microcapsules had a homogeneous, spherical structure with a smooth surface, indicating uniform polymer organization after crosslinking with calcium ions. The observed differences in capsule sizes were correlated with their degradation behavior, with smaller capsules exhibiting faster erosion due to their higher surface area-to-volume ratio. Additionally, minor discoloration inside the microcapsules suggested variations in the distribution of doxorubicin, potentially affecting its release kinetics.

Importantly, the quantitative drug release study demonstrated that the amount of albumin incorporated into the alginate matrix has a decisive impact on the 24-h release behavior of doxorubicin. Higher albumin content significantly reduced DOX release, indicating stronger drug–protein interactions and a denser internal network capable of retaining the drug more efficiently. This tunable release profile underscores the potential of alginate–albumin microcapsules as controlled drug delivery systems.

In summary, this work demonstrates that alginate–albumin microcapsules represent a versatile and tunable platform for biomedical applications. By adjusting the composition of the polymer matrix, particularly the albumin content, it is possible to modulate key functional properties of the carriers, including structural stability, degradation behaviour, and drug release performance. The combined physicochemical, spectroscopic, and microscopic analyses provide a solid foundation for understanding the interactions within the polymer network and their influence on the overall functionality of the microcapsules. These findings highlight the potential of such hybrid biopolymer systems for the development of controlled drug delivery strategies, laying the groundwork for future studies aimed at optimizing internal architecture, mechanical robustness, and therapeutic efficacy.

## Figures and Tables

**Table 1:** Summary of the results of the diameter of the tested samples (a) 50 mL series (b) 100 mL series.

Sample Name	Diameter [µm]	Sample Name	Diameter [µm]
2Alb_50mL	1239	2Alb_100mL	2168
4Alb_50mL	1262	4Alb_100mL	2683
6Alb_50mL	1334	6Alb_100mL	2442
8Alb_50mL	1433	8Alb_100mL	2579

**Table 2:** The composition of the substrates used for protein synthesis.

Sample Number	Sodium Alginate [mL]	Albumin [mg/mL]	Doxorubicin [mg]	Sample Name
1	20	2	2.5	2Alb_50mL/2Alb_100mL
2		4		4Alb_50mL/4Alb_100mL
3		6		6Alb_50mL/6Alb_100mL
4		8		8Alb_50mL/8Alb_100mL

## Data Availability

The original contributions presented in this study are included in the article. Further inquiries can be directed to the corresponding authors.
